# An Optimum Population

**Published:** 1919-05-24

**Authors:** 


### AN OPTIMUM POPULATION.

The war is over. It is too soon for its history to be written with clear and unbiased judgment. That is work for historians of the next generation. Then the complex causes which led to the great and terrible effect that has passed will be seen in perspective and weighed and duly appreciated. It is likely that of the causes one will be held to be the natural increase of the German population in the territory occupied by them. They had to find outlet and suitable settlement for the excess resulting, because the lands around were already populated nearly to the full and effectively occupied by other races with other thoughts and ways; and because the more distant temperate lands, though not fully populated, yet were held and required for their increasing peoples by nations not prepared to forgo their rights. If this was the cause of war history may again repeat itself. Outside Europe, China, Japan, and India are crowded countries. America is filling fast. Africa has still vast spaces, but much of the land does not allow existence in it of folk of temperate climes. The world is getting fully peopled.

### The World in Future.

It is many years since Malthus wrote. His views were not acceptable, but they were based on truths. Chiefly he was concerned with food supply, and was prepared for a time of miserable human experience, and that increasingly miserable. But mankind has begun to learn again that this world is a beautiful place and good to live in. The sad old view that it is but a proving place and purgatory, and this life only something to regret, the pleasures of it snares, and the wondrous gifts and faculties of man things to be sternly repressed, is fading away. Man in the future will take it to be right to enjoy his present to the full, will learn also how rightly to enjoy, and casting aside the wicked old terrorisms of the priests will face the unknowable other side of the veil with courage and the cheerfulness which comes of confidence in the Author of the Universe, Who meant all creatures to be happy or non-existent. Life must be something more than existence on the starvation line, and for the future there must be abounding buoyant hope, hope not only of the individual for his own future, but certain confident hope for the future of the generations he shall not see but shall hope to shape. It may be, it will be, the individual's rule for living that he must do all he can, not for himself and his salvation only, but for the generations of his race that shall succeed him. If such be his rule man must consider what life ought to be, and whatsoever life ought to be, to live it as it ought to be depends on space. Some have dreamt of garden cities, some of three acres and a cow, some of small self-supporting communities each ruling itself without regard to others; but consideration will show that, whatever the view, the life cannot be lived if the population on the ground increase beyond a certain number. Probably no one view is the only right view. The diversities of mankind demand the possibility of many ways of living. Some prefer and are happiest in great, spacious, beautiful cities, some in peaceful rural scenes, some in vast and wild spaces with solitude. For the full development of the potentialities of man it may be necessary that all these conditions of living should be possible. But if population increases beyond a certain number none of these can be. The spacious cities become agglomerations of crowded streets, the rural beauty is narrowed down to hedgeless fields bereft of woodland, heath and coppice, the wilds become tame and crowded, and mankind in crowds will be the only kind of mankind.

### Our Best Evolution.

Will that be our best evolution? It may be wise for mankind to consider what may be the best human population for this planet, for a best population there certainly must be—a best population for every one of the divisions into which the world is divided and a best population for the world as a whole. It may be that some of the most fully developed and populated countries have already attained to or even passed the limit that is best. It may be that some districts, though capable of population, are best reserved as playgrounds or solitude to give the chance of change to dwellers in the crowded cities. A best population is not only the maximum number that can exist in an area, but the maximum number that can live a full life and keep healthy and happy, sane of body and mind as when the Creator looked on what He had created and saw that it was good. The population must be good again; good all through and evolving to better and better. This can be, for man by virtue of his reason is master of the destiny of his kind, is capable of shaping the form and attributes of future generations. It is for man to decide whether he will assist the Author of the Universe to raise the species to higher and higher planes, or to thwart beneficent purpose and doom the human race to decay and extinction.

